# Exploring the pathways between physical activity, love for nature and eco-friendly behavior in children

**DOI:** 10.3389/fpsyg.2025.1710555

**Published:** 2026-01-12

**Authors:** Yannis Theodorakis, Aristea Karamitrou, Charalampos Krommidas, Aikaterini Violatzi, Maria Angeli, Mary Hassandra, Nikos Comoutos

**Affiliations:** Department of Physical Education and Sport Sciences, University of Thessaly, Trikala, Greece

**Keywords:** children, eco-friendly behaviors, environmental attitudes, love for nature, physical activity, sustainability, Theory of Planned Behavior

## Abstract

**Background:**

This study aims to examine the relationships between involvement in Physical Activity (PA), love for nature, attitudes, subjective norms, and Perceived Behavioral Control (PBC) toward, and intentions to perform eco-friendly behaviors. A general feeling of connectedness to nature and related attitudes has been shown to promote eco-friendly behaviors. However, the specific pathways through which this connectedness influences behavior have not been systematically examined.

**Method:**

Participants were 672 students aged 9–13 years old (*M* = 11.28, *SD* = 0.68) who completed several instruments measuring PA, the love and care for nature, and, based on the Theory of Planned Behavior (TPB), attitudes, subjective norms, PBC, and intentions to perform eco-friendly behaviors. Also, a list of pro-environmental behaviors, PA related behavior and energy-saving behaviors were distributed.

**Results:**

Serial-parallel mediation analyses showed that PA significantly (positively) predicted love for nature, which in turn significantly (positively) predicted attitudes, subjective norms, and PBC. These three TPB constructs significantly (positively) predicted students’ intentions to act in eco-friendly ways. In turn, intentions significantly (positively) predicted all three categories of eco-friendly behaviors. The models revealed significant direct and indirect effects, supporting a robust pathway from PA to sustainable behaviors via psychological and attitudinal mediators.

**Conclusion:**

The findings underscore the value of the TPB in understanding how PA and a love for nature contribute to the development of positive attitudes, perceived social norms, and control beliefs related to eco-friendly behaviors among students. This study highlights the importance of fostering both PA and a connection with nature in educational settings, as these factors play a pivotal role in shaping students’ attitudes, subjective norms, PBC, intentions, and actual engagement in sustainable, eco-friendly actions. Future interventions to promote environmental responsibility among youth should integrate activities that foster both nature connectedness and active lifestyles within urban settings.

## Introduction

In recent years, the value of physical activities in environmental protection has become increasingly recognized. Cycling and walking can help combat obesity, physical inactivity, and air pollution. These factors together contribute to over 1.5 million deaths annually in Europe (World Health Organization, 2022). A report from the World Health Organization (WHO) highlights that those policies promoting these activities can improve public health, reduce greenhouse gas emissions, and support the achievement of Sustainable Development Goals by mitigating climate change and enhancing environmental quality (World Health Organization, 2022). Furthermore, a key challenge in promoting Physical Activity (PA) lies in ensuring that the built environment supports and facilitates active living. While enhancing environmental factors that influence PA is crucial, these changes take time to implement. Identifying more tailored approaches for PA recommendations could further support the promotion of healthier lifestyles (Giles et al., 2021).

A systematic review has shown that climate change impacts PA on a global scale, with PA playing a dual role in both mitigating and exacerbating climate change (Bernard et al., 2021). While PA is widely recognized for its substantial health benefits, certain forms particularly nature sports can negatively affect the environment. This is largely due to the carbon emissions associated with spectator travel, as well as the high energy demands of infrastructure and maintenance for facilities like indoor swimming pools, ski resorts, and golf courses. In contrast, activities such as active transportation and related urban adaptations, outdoor exercising, and gardening not only support individual health but also contribute positively to planetary health. Given this complex interplay, it is essential that efforts to promote PA are aligned with the principles of planetary health (Abu-Omar et al., 2023).

Pro-environmental behaviors and energy-saving behaviors refers to actions aimed at reducing environmental harm or actively contributing to the restoration of the natural environment. These behaviors can be carried out in both private and public spheres. In the private domain, actions include activities such as recycling, making environmentally conscious purchases, and conserving water and energy. In the public domain, pro-environmental behavior may involve encouraging others to protect the environment or participating in environmental groups (Steg and Vlek, 2009). A relevant study examined the relationship between pro-environmental behaviors, visits to natural spaces, connection with nature and PA. It found that men who engaged in higher levels of PA and walked more often were more likely to prefer biking as a mode of transportation. Additionally, those who visited nature more frequently showed greater involvement in unpaid activities aimed at preserving the environment. The study also highlighted an association between engaging in recommended levels of PA and increased pro-environmental behaviors, including recycling, purchasing seasonal and locally grown products, adopting green travel practices, volunteering for environmental causes, and encouraging others to adopt pro-environmental behaviors (Teixeira et al., 2022).

Examining the impact of exercising in natural settings on both health and environmental consciousness reveals that participation in sports and recreational activities is closely linked to wellbeing and eco-conscious behaviors. These activities play a vital role in enhancing environmental awareness and fostering sustainable attitudes (Niu et al., 2024). Nature-based physical activities, in particular, are instrumental in promoting and protecting wellbeing. To fully leverage their benefits, opportunities for such activities should be more effectively integrated across various sectors, including education, sports, public health, and urban planning (Turecek et al., 2025). Research has shown that PA, especially when shared with parents, is positively correlated with a stronger connection to nature (Puhakka et al., 2018). Furthermore, engaging in nature-based physical activities can enhance two distinct types of psychological wellbeing: hedonic wellbeing and eudaimonic wellbeing (Jenkins et al., 2022). The profound love and reverence for nature are cultivated through immersive, direct experiences within natural environments. Promoting such activities within communities stands as a compelling strategy for fostering a deeper sense of environmental stewardship and, by extension, reinforcing our commitment to the protection of the natural world. This is rooted in the understanding that internalized motivations are pivotal in nurturing and sustaining consistent, environmentally responsible behavior. In light of this, the exploration of internalized motivations particularly the human sense of interconnectedness and community with nature has become a focal point of empirical research (Perkins, 2010). Additionally, both attitudes toward nature and environmental consciousness are key factors in deepening our understanding of the environment (Baierl et al., 2024).

Environmental risks contribute significantly to the global disease burden, accounting for nearly a quarter of all health issues worldwide. Air pollution alone is responsible for almost 7 million deaths each year (World Health Organization, 2025). In Europe, 78% of people acknowledge that environmental problems have a direct impact on their daily lives and health (European Union, 2024). Individuals experiencing severe climate-related worry tend to engage in pro-environmental behaviors and report a need for support, particularly in relation to worry management strategies and the adoption of sustainable lifestyle practices (Lenhard et al., 2024). Given this, the attitudes and behaviors of young people toward the environment are crucial in shaping effective protection and conservation efforts. By nurturing positive environmental development in youth, the next generation will be empowered to actively engage in environmental conservation, ensuring a future where sustainable practices and positive attitudes toward the environment flourish (Bøhlerengen and Wiium, 2022).

The Sustainable Development Goals outline actions to protect our environment and combat climate change. Key actions for a healthier planet include energy-saving at home, changing your home’s energy source, walking, biking, or taking public transport, switching to an electric vehicle, considering your travel choices, reducing, reusing, repairing, recycling, eating more vegetables, wasting less food, planting native species, cleaning up your environment, making your money count, and speaking up to encourage others to act (United Nations, 2025). For example, promoting active travel should be a cornerstone of strategies to meet net-zero carbon targets, particularly in urban areas, while also improving public health and the quality of urban life (Brand et al., 2021).

Energy conservation is a crucial pro-environmental behavior widely recognized as essential for safeguarding the environment. Understanding the factors that drive pro-environmental behavior among young people is vital for shaping a sustainable future. Individuals’ behaviors are influenced by their attitudes, and one prominent framework for exploring pro-environmental intentions and actions is the Theory of Planned Behavior (TPB). Studies have shown a strong alignment between the standard TPB model and empirical data, reinforcing its effectiveness in understanding pro-environmental behaviors (de Leeuw et al., 2015). Meta-analyses examining the TPB across various contexts suggest that, particularly in developed and individualistic countries, intentions to engage in pro-environmental behavior are more likely to be realized in actual actions. In these settings, attitudes toward the environment are closely linked to environmental intentions. Furthermore, in developed countries, perceived behavioral control (PBC) is partially associated with environmental intentions (Morren and Grinstein, 2016). Bibliometric analyses have provided additional valuable insights into the application of the TPB within environmental science. These findings serve as a comprehensive resource for researchers, offering new perspectives and directions for future studies on behavior related to environmental protection, and highlighting the broader applicability of the TPB in this domain (Si et al., 2019).

Moreover, meta-analytic research has uncovered key factors that influence consumer decision-making when purchasing green products. These factors—attitude, subjective norms, PBC, and environmental consciousness—are all positively associated with green purchase intentions. Among these, attitude exerts the strongest influence on purchase intention, followed by PBC, subjective norms, and, finally, environmental consciousness (Panda et al., 2024). Finally, in the context of road-running events, participants who are more attuned to environmental issues tend to exhibit more favorable attitudes toward promoting environmental protection and are more likely to be influenced by subjective norms. To encourage greater participation in such events, it is essential to implement environmental protection measures that enhance public awareness and understanding of environmental issues in these settings (Pai et al., 2024).

Using data from a large international survey conducted in 11 countries, a study by Miller et al. (2022) found that environmental attitudes are a significantly stronger predictor of pro-environmental behaviors than other factors. While evidence continues to grow regarding the influence of TPB components and nature connectedness, few studies have integrated these variables within a comprehensive mediation model. Building on this emerging framework and the established links between nature connectedness and pro-environmental attitudes the present study aims to examine how PA may promote sustainable behaviors through these interconnected psychological mechanisms. More specifically, this study aims to examine the relationships between PA, a love and care for nature, and pro-environmental behaviors, with the mediating role of the TPB factors: attitudes, subjective norms, PBC, and the intention to engage in eco-friendly actions. Physical activities such as walking and cycling for transportation, or opting for stairs instead of elevators, are examples of environmentally friendly behaviors that reflect a love for nature. Additionally, this love for nature strengthens positive attitudes toward the environment. Ultimately, positive attitudes toward the environment, along with pro-environmental actions, shape PBC, subjective norms, and, in turn, the intention to adopt sustainable behaviors.

An additional issue warranting investigation within the scope of the present study concerns gender differences in perceptions of and responses to climate change. The impacts of climate change vary substantially across populations, with gender playing a critical role in shaping how individuals perceive, interpret, and respond to climate-related risks (Verner et al., 2026). Empirical evidence indicates that women report statistically significantly higher levels of concern about climate change than men in a number of countries; however, this gender gap is not observed uniformly across the globe. Research suggests that gender differences in climate change attitudes are statistically significant primarily in relatively affluent societies, with the magnitude of the gap increasing alongside levels of economic prosperity. Moreover, greater exposure to climate-related risks appears to intensify gender differences in expressed concern, though not necessarily in perceptions of the overall seriousness of climate change (Knight and Givens, 2021; Bush and Clayton, 2023). Given that the present study focuses on younger age groups, it is particularly important to examine whether such gender-based differences emerge early in life, as early-formed attitudes and perceptions may play a formative role in shaping future environmental awareness, concern, and pro-environmental behavior. Finally, the necessity of the present research is further underscored by the lack of empirical studies focusing on children aged 9–13, as existing research has predominantly examined adolescents and, more extensively, adult populations.

While prior research has primarily examined the direct relationships among the aforementioned variables, limited attention has been given to the combined influence of multiple mediators. Relying solely on a single-mediation model may oversimplify the complex mechanisms underlying these relationships. To address this gap, the present study develops a comprehensive multiple, serial-parallel mediation model to empirically explore how love for nature, attitudes, subjective norms, PBC, and intentions mediate the relationship between PA and eco-friendly behaviors.

Based on previous research findings indicating that PA is positively related to a stronger connection to nature (Puhakka et al., 2018; Jenkins et al., 2022) and to a variety of pro-environmental behaviors, both in the private and public domain (Teixeira et al., 2022); the positive relationship between love for nature and pro-environmental attitudes (Baierl et al., 2024); and the positive relationships among TPB factors (attitudes, subjective norms, PBC, and intention toward eco-friendly actions) with eco-friendly actions, we hypothesized that: PA will significantly and positively predict students’ love and care for nature (M_1_), which in turn will significantly and positively predict students’ attitudes (M_2_), subjective norms (M_3_), and PBC (M_4_) toward eco-friendly behaviors. Students’ attitudes (M_2_), subjective norms (M_3_), and PBC (M_4_), will each significantly and positively predict students’ intention to perform eco-friendly behaviors (M_5_). Lastly, students’ intention to perform eco-friendly behaviors (M_5_) will significantly and positively predict each of the three eco-friendly behaviors subscales.

**Figure 1:**
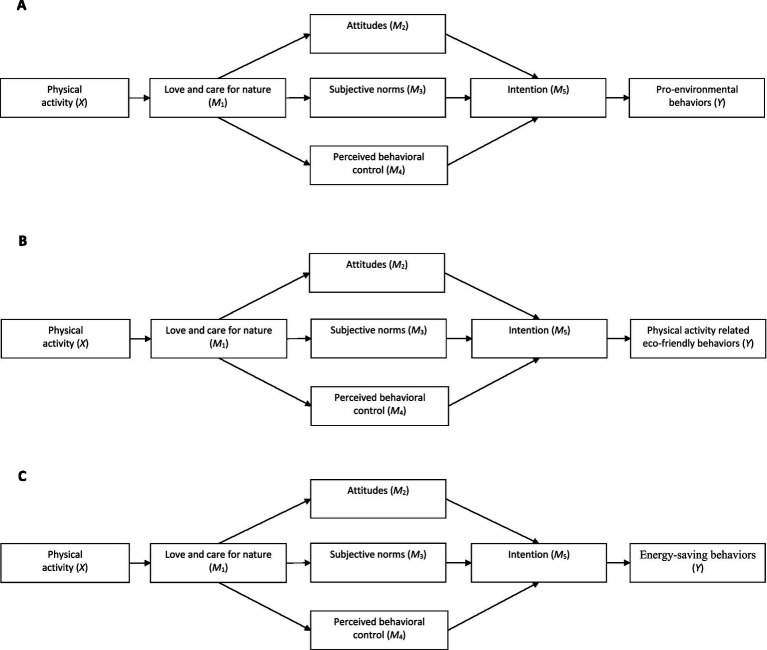
Conceptual serial-parallel mediation models linking the students’ physical activity to eco-friendly behaviors subscales via the serial-parallel mediators: Love and care for nature (M1), attitudes (M2), subjective norms (M3), perceived behavioral control (M4), and intention (M5). Covariate paths (gender) are omitted for clarity. **(A)** Conceptual serial-parallel mediation model linking the students’ physical activity to pro-environmental behaviors via the serial-parallel mediators. **(B)** Conceptual serial-parallel mediation model linking the students’ physical activity to physical activity related eco-friendly behaviors via the serial-parallel mediators. **(C)** Conceptual serial-parallel mediation model linking the students’ physical activity to energy-saving behaviors via the serial- parallel mediators.

The current study proposed and examined three path models of the relationships between students’ PA and eco-friendly behaviors subscales, with love and care for nature, attitudes, subjective norms, PBC, and intentions toward eco-friendly behaviors acting as serial-parallel mediators (see Figures 1A–C). The proposed models also incorporated gender as a covariate to provide a comprehensive analysis of its unique variance on each mediator and outcome, as it has been found to be associated with some of the examined variables in the previous research (Gifford and Nilsson, 2014; Martin et al., 2020).

## Materials and methods

### Participants

In this cross-sectional, population-based study, participants were recruited through school administrators. The target population comprised children enrolled in elementary schools located in central Greece. Sample size was determined *a priori* via power analysis to ensure adequate statistical power. A quota sampling strategy was employed, stratified by age, gender, and geographic region, to enhance the representativeness of the sample.

The final sample consisted of 672 elementary school students, aged 9–13 years (*M* = 11.28, *SD* = 0.68), drawn from 21 school units across five distinct areas of central Greece. In terms of gender distribution, 309 participants identified as boys and 354 as girls, while nine students did not report their gender. Regarding school grade, 231 students were enrolled in the 5th primary class and 437 in the 6th primary class, with data missing for four participants. As for ethnicity, the majority of students (*n* = 602) identified as Greek, 25 as Albanian, one as Bulgarian, and six reported other ethnicities; ethnicity data were unavailable for 38 students.

### Measures

#### Physical activity (PA)

PA was measured using a self-reported PA scale to record the frequency of children’s engagement in moderate-to-vigorous PA per week (Booth et al., 2001), which has already been applied in studies with adolescents in Greece (Maltagliati et al., 2023).

#### Love and care for nature

To assess students’ love and care for nature, the 15-item Love and Care for Nature (Perkins, 2010) was administered. Example items include: “I feel joy just being in nature.,” “I feel a deep love for nature.” Respondents were asked to respond to each of the 15 items on a 7-point Likert-type scale ranging from 1 (*strongly disagree*) to 7 (*strongly agree*).

#### TPB variables

Participants were then asked to complete scales assessing the variables of the TPB and particularly eco-friendly attitudes, subjective norms, PBC, and intentions. The scales were constructed according to Ajzen (2006) recommendations and their adaptation to the Greek language (Theodorakis, 1994). Initially, a written description was given of what eco-friendly behaviors are, along with relevant examples of such behaviors. Subsequently, the related scales of the TPB variables followed, as analyzed below:

Attitude toward eco-friendly behaviors: To assess attitudes toward eco-friendly behavior, participants evaluated the common stem, “For me, performing eco-friendly behaviors on a regular basis during the next 6 months would be” using five 7-point bipolar adjective scales, such as “useless-useful,” “unpleasant-pleasant,” “good-bad,” “happy-sad,” and “pretty-ugly.”

PBC: Participants rated the following two items on 7-point scales: “For me, performing eco-friendly behaviors on a regular basis in the next 6 months would be” (ranging from “very difficult” to “very easy”), and “I feel that I’m able to perform pro-environmental behaviors on a regular basis in the next 6 months” (ranging from “definitely not” to “yes, definitely”).

Subjective norms: Subjective norms to perform eco-friendly behaviors were assessed using two items: “In general, people who are close to me expect me to adopt eco-friendly behaviors on a regular basis during the next 6 months” and “People who are important to me will perform eco-friendly behaviors on a regular basis during the next 6 months.” Participants responded to each item on a 7-point scale ranging from “definitely not” to “yes, definitely.”

Intentions: Intentions to perform eco-friendly behaviors were assessed by computing the mean response to the following three items: “I intent/I will try/I am determined/to perform eco-friendly behaviors on a regular basis during the next 6 months.” Responses were provided on 7-point scales ranging from “definitely not” to “yes, definitely.

#### Eco-friendly behaviors

To measure eco-friendly behaviors, a set of 20 items representing three distinct subscales was utilized. The first subscale comprised 10 pro-environmental behavior items (e.g., “I leave the water running while I brush my teeth,” “At school, I put my trash in the proper recycling bin”) (de Leeuw et al., 2015). The PA-related energy-saving behaviors subscale was assessed using six items (e.g., “I go to school by bike or on foot,” “At home, I prefer to use the stairs instead of the elevator,” “In general, for short distances, I prefer to walk, both in winter and summer”). The items were adapted from the environmentally significant behaviors associated to physical activity (Eriksson and Linde, 2024) and the pro-environmental behavior scale (Ojala, 2012). The energy-saving behaviors subscale was assessed using four items (e.g., “I switch off the lights even if I’m not using them for a short time,” “I do not switch on the lights if natural light is sufficient”) (Suntornsan et al., 2022). Participants were asked to indicate how often they perform each of the above behaviors on a 7-point Likert-type scale ranging from 1 (never) to 7 (very often). For a complete list of items please see Supplementary file 1.

### Procedure

In this study we followed a cross-sectional design. Initially, the research approval by the Bioethics Committee of the authors’ University was obtained (Ref: 09/Date: 06.02.2024). Then, parents/guardians and participants were fully informed about the study’s purpose and that their participation was entirely voluntary. After participants and their parents/guardians provided written consent, data collection was conducted during a physical education class by an experienced researcher. The completion of the questionnaires lasted about 10 min.

### Data analysis

All analyses were conducted using SPSS version 24.0 and the PROCESS-Macro for SPSS version 4.2, an observed variable path analysis modeling tool that is based on regression (Hayes, 2022). PROCESS macro is widely used through the social, business, and health sciences to perform mediation, moderation and conditional process analysis. For this analysis, three custom models were constructed, to test the proposed serial-parallel mediation models shown in Figures 1A–C. As Figures 1A–C depict, in each model, we entered the amount of PA as the independent variable (X), one of the energy-saving behaviors subscales as the outcome variable (Y), and love and care for nature, attitudes to save energy, subjective norms to save energy, PBC to save energy, and intention to save energy as serial-parallel mediators (M_1_, M_2,_ M_3,_ M_4,_ M_5_ respectively). Also, we included gender as a covariate (antecedent variable) in the models of all mediators (M_1_–M_5_), as well as in the models of all outcome variables (Y).

The PROCESS macro utilizes a bootstrapping approach to evaluate the confidence interval (CI) of the size of particular model-specified indirect effects (*ab*). Bootstrapping entails resampling the original sample thousands of times (e.g., 10,000 times as tested herein) and computing the indirect effect (*ab*) in each sample to create a sampling distribution of the indirect effect. This distribution is used to construct a bootstrap CI. A relatively new approach to mediation analysis, Hayes’s (2022) procedure is superior to the Sobel test and the commonly used Baron and Kenny’s causal step approach in terms of statistical power, control over Type I error, and testing multiple mediators. Also, in contrast to the Sobel test, it does not require the assumption of normality of the sampling distribution of *ab*, which is often not the case (Preacher and Hayes, 2004). In the examination of multiple mediator models, PROCESS macrο allows for the estimation of total, direct, total indirect, and specific indirect effects through each mediator (whilst controlling for effects of all the other mediators). According to Hayes (2022) within multiple mediation models, a significant total indirect effect (i.e., the sum of specific indirect effects) is not necessary in order to examine the specific indirect effects, as a specific indirect can be significant even though the total indirect effect is not. We can conclude that a specific indirect effect or a total indirect effect is statistically significant at *p* < 0.05 when the 95% bootstrap CI does not contain zero.

Finally, before the main analysis, the data were screened, and descriptive statistics, scale reliabilities, and Pearson’s correlations were calculated for all study variables.

## Results

### Descriptive statistics, reliabilities, and correlations

Descriptive statistics, internal reliability scores, and correlations for key examined variables are presented in Table 1. All scales showed acceptable internal reliability (Cronbach’s *α*). Correlation analyses revealed that all variables correlated significantly and positively with each other (from low to moderately), except the correlations between PA and six of the variables, which were non-significant (*p* > 0.05).

**Table 1:** Descriptive statistics, Cronbach’s alpha, and correlations for all study variables.

Variables	*M*	*SD*	*a*	1	2	3	4	5	6	7	8	9
1. Physical activity	3.33	1.18	–	–								
2. Love and care for nature	5.47	0.88	0.91	0.10**	–							
3. Attitudes toward eco-friendly behaviors	5.90	0.97	0.73	0.09*	0.23***	–						
4. Subjective norms to perform eco-friendly behaviors	5.81	1.23	0.79	0.03	0.25***	0.33***	–					
5. Perceived behavioral control	5.13	1.29	0.71	−0.01	0.24***	0.29***	0.50***	–				
6. Intention to perform eco-friendly behaviors	5.26	1.28	0.74	0.07	0.25***	0.43***	0.53***	0.59***	–			
7. Pro-environmental behaviors	4.53	1.04	0.67	−0.01	0.29***	0.14**	0.18***	0.20***	0.26***	–		
8. Physical activity related eco-friendly behaviors	4.80	1.19	0.61	0.07	0.18***	0.10*	0.11**	0.13**	0.12**	0.36***	–	
9. Energy-saving behaviors	5.09	1.26	0.61	−0.02	0.24***	0.12**	0.15***	0.11**	0.18***	0.44***	0.28***	–

Physical activity (possible range 0–5); Love and care for nature (possible range 1–7); Attitudes (possible range 1–7); Subjective norms (possible range 1–7); Perceived behavioral control (possible range 1–7); Intention (possible range 1–7); Pro-environmental behaviors (possible range 1–7); Physical activity related behaviors (possible range 1–7); Energy-saving behaviors (possible range 1–7). *p < 0.05. **p < 0.01. ***p < 0.001.

### Serial-parallel mediation models analyses

**Figure 2:**
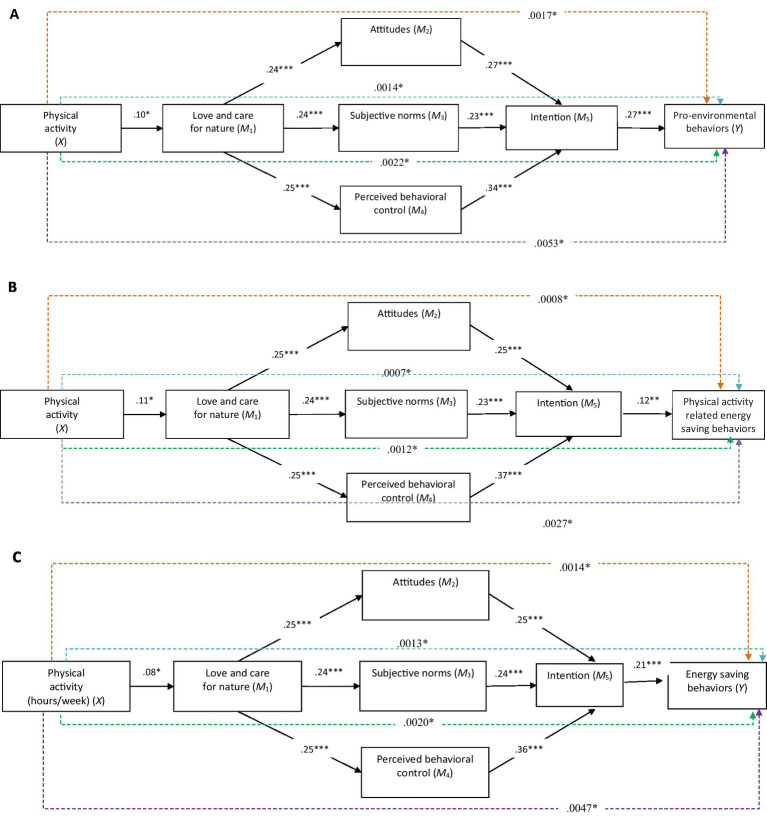
The statistical diagrams of the serial-parallel mediation models linking the students’ physical activity to eco-friendly behaviors subscales via the serial-parallel mediators: love and care for nature (M_1_), attitudes to save energy (M_2_), subjective norms to save energy (M_3_), perceived behavioral control to save energy (M_4_), and intention to save energy (M_5_). Covariate paths (gender) are omitted for clarity. Standardized path coefficients are displayed for ease of comparison. Solid line = significant direct effect; orange dashed line = significant specific indirect effect via love and care for nature, attitudes, and intention; light blue dashed line = significant specific indirect effect via love and care for nature, subjective norms, and intention; green dashed line = significant specific indirect effect via love and care for nature, perceived behavioral control, and intention; purple dashed line = significant total indirect effect. *p < 05. ***p < 0.0.001.

Results of the proposed serial-parallel mediation models analyses are displayed in Figures 2A–C, and summarized in Table 2 (direct effects), and Table 3 (indirect effects). Tables 2, 3 include both unstandardized coefficients to aid interpretation of effects relative to response scales, and standardized coefficient for comparison across predictors. As can be seen in Table 2, in all of the three models, the PA with gender as a covariate significantly explained 2% of variance in love and care for nature (M_1_). Love and care for nature (M_1_) with gender (covariate) significantly explained: 6% of the variance in attitudes toward eco-friendly behaviors (M_2_) and 7% of the variance in subjective norms (M_3_) in all three models, 7% of the variance in PBC towards eco-friendly behaviors (M_4_) in the serial-parallel mediation model linking students’ PA to pro-environmental behaviors, and 8% of the variance in PBC to save energy (M_4_) in the serial-parallel mediation models linking the amount of students’ PA to PA related energy-saving behaviors and energy-saving behaviors. Attitudes towards eco-friendly behaviors (M_2_), subjective norms (M_3_), PBC (M_4_), and the covariate (gender) significantly explained 42% of the variance in intention to perform eco-friendly behaviors (M_5_) in all of the three models. Finally, intention to perform eco-friendly behaviors (M_5_) with the covariate (gender) significantly accounted for 7% of the variance in pro-environmental behaviors, 2% of the variance in PA related energy-saving behaviors, and 5% of the variance in energy-saving behaviors.

**Table 2:** Regression models statistics and direct effects of key variables and covariate (gender) for the serial-parallel mediation models linking students’ physical activity to pro-environmental behaviors subscale via the serial-parallel mediators: Love and care for nature (M_1_), attitudes (M_2_), subjective norms (M_3_), perceived behavioral control (M_4_), and intention (M_5_).

Predictor	Outcome: Love and care for nature (M_1_)			Outcome: Attitudes (M_2_)			Outcome: Subjective norms (M_3_)			Outcome: Perceived behavioral control (M_4_)			Outcome: Intention (M_5_)			Outcome: Pro-environmental behaviors (Y)		
	*B*	*SE*	*β*	*B*	*SE*	*β*	*b*	*SE*	*β*	*B*	*SE*	*β*	*b*	*SE*	*β*	*B*	*SE*	*β*
Serial-parallel mediation model linking the students’ physical activity to pro-environmental behaviors																		
Physical Activity (X)	0.07*	0.03	0.10													.		
Love and care for nature (M_1_)				0.26***	0.05	0.24	0.31***	0.06	0.24	0.35***	0.06	0.25						
Attitudes (M_2_)													0.35***	0.05	0.27			
Subjective norms (M_3_)													0.24***	0.04	0.23			
Perceived behavioral control (M_4_)													0.33***	0.04	0.34			
Intention (M_5_)																0.23***	0.04	0.27
Gender	0.18*	0.08	0.10	- 0.01	0.08	−0.01	0.19*	0.10	0.08	0.19	0.11	0.08	−0.06	0.08	−0.03	0.10	0.09	0.05
Constant	4.96***	0.18		4.53***	0.27		3.88***	0.33		2.93***	0.36		0.20	0.31		3.17***	0.24	
	*R^2^* = 0.02 *F* (2, 518) = 4.42, *p* = 0.013			*R^2^* = 0.06 *F* (2, 518) = 16.23, *p* < 0.001			*R^2^* = 0.07 *F* (2, 518) = 18.42, *p* < 0.001			*R^2^* = 0.07 *F* (2, 518) = 19.45, *p* < 0.001			*R^2^* = 0.42 *F* (4, 516) = 92.57, *p* < 0.001			*R^2^* = 0.07 *F* (2, 518) = 20.63, *p* < 0.001		
Serial-parallel mediation model linking the students’ physical activity to physical activity related energy saving behaviors																		
Physical Activity (X)	0.08*	0.03	0.11													.		
Love and care for nature (M_1_)				0.27***	0.05	0.25	0.30***	0.05	0.24	0.36***	0.06	0.25						
Attitudes (M_2_)													0.32***	0.05	0.25			
Subjective norms (M_3_)													0.24***	0.04	0.23			
Perceived behavioral control (M_4_)													0.35***	0.04	0.37			
Intention (M_5_)																0.12**	0.04	0.12
Gender	0.18*	0.08	0.10	- 0.01	0.08	0.00	0.19*	0.10	0.09	0.23*	0.11	0.09	−0.05	0.08	−0.02	0.10	0.11	0.04
Constant	4.91***	0.18		4.46***	0.28		3.91***	0.32		2.86***	0.36		0.27	0.31		4.06***	0.28	
	*R^2^* = 0.02 *F* (2, 517) = 5.20, *p* = 0.006			*R^2^* = 0.06 *F* (2, 517) = 17.05, *p* < 0.001			*R^2^* = 0.07 *F* (2, 517) = 18.63, *p* < 0.001			*R^2^* = 0.08 *F* (2, 517) = 21.25, *p* < 0.001			*R^2^* = 0.42 *F* (4, 515) = 92.87, *p* < 0.001			*R^2^* = 0.02 *F* (2, 517) = 4.11, *p* = 0.017		
Serial-Parallel Mediation Model Linking the Students’ Physical Activity to Energy-Saving Behaviors																		
Physical activity (X)	0.08*	0.03	0.10													.		
Love and care for nature (M_1_)				0.28***	0.05	0.25	0.31***	0.05	0.24	0.36***	0.06	0.25						
Attitudes (M_2_)													0.31***	0.05	0.25			
Subjective norms (M_3_)													0.26***	0.04	0.24			
Perceived behavioral control (M_4_)													0.34***	0.04	0.36			
Intention (M_5_)																0.21***	0.04	0.21
Gender	0.19*	0.08	0.11	- 0.01	0.08	−0.01	0.21*	0.10	0.09	0.23*	0.11	0.09	−0.05	0.08	−0.02	0.05	0.10	0.02
Constant	4.92***	0.18		4.42***	0.27		3.81***	0.33		2.82***	0.36		0.25	0.30		3.91***	0.27	
	*R^2^* = 0.02 *F* (2, 528) = 5.11, *p* = 0.006			*R^2^* = 0.06 *F* (2, 528) = 18.19 *p* < 0.001			*R^2^* = 0.07 *F* (2, 528) = 20.17, *p* < 0.001			*R^2^* = 0.08 *F* (2, 528) = 21.95 *p* < 0.001			*R^2^* = 0.42 *F* (4, 526) = 96.69, *p* < 0.001			*R^2^* = 0.05 *F* (2, 528) = 12.50, *p* < 0.001		

*b* = Unstandardized coefficients; *β* = Standardized coefficients; *SE* = Standard error. *p < 0.05. **p < 0.01. ***p < 0.001.

**Table 3:** Indirect effects (specific and total) of the students’ physical activity to pro-environmental behaviors subscale via the serial-parallel mediators: love and care for nature (M_1_), attitudes (M_2_), subjective norms (M_3_), perceived behavioral control (M_4_), and intention (M_5_).

Specific indirect effects	*b*	*SE* of *b*	95% BC bootstrap CI for *b*	*β*	*SE* of *β*	95% BC bootstrap CI for *β*
Serial-parallel mediation model linking students’ physical activity to pro-environmental behaviors						
Physical activity → love and care for nature → attitudes → intention → pro-environmental behaviors	0.0015*	0.0009	[0.0001, 0.0038]	0.0017*	0.0010	[0.0002, 0.0044]
Physical activity → love and care for nature → subjective norms → intention → pro-environmental behaviors	0.0012*	0.0007	[0.0002, 0.0032]	0.0014*	0.0009	[0.0002, 0.0038]
Physical activity → love and care for nature → perceived behavioral control → intention → pro-environmental behaviors	0.0019*	0.0010	[0.0002, 0.0046]	0.0022*	0.0012	[0.0003, 0.0053]
Total indirect effect	0.0046*	0.0025	[0.0004, 0.0104]	0.0053*	0.0029	[0.0004, 0.0119]
Serial-parallel mediation model linking students’ physical activity to physical activity related energy-saving behaviors						
Physical activity → love and care for nature → attitudes → intention → physical activity related energy-saving behaviors	0.0008*	0.0006	[0.0001, 0.0026]	0.0008*	0.0006	[0.0001, 0.0026]
Physical activity → love and care for nature → subjective norms → intention → physical activity related energy-saving behaviors	0.0007*	0.0005	[0.0001, 0.0024]	0.0007*	0.0005	[0.0001, 0.0023]
Physical activity → love and care for nature → perceived behavioral control → intention → physical activity related energy-saving behaviors	0.0012*	0.0008	[0.0002, 0.0036]	0.0012*	0.0008	[0.0002, 0.0035]
Total indirect effect	0.0027*	0.0017	[0.0004, 0.0077]	0.0027*	0.0017	[0.0004, 0.0076]
Serial-parallel mediation model linking the students’ physical activity to energy-saving behaviors						
Physical activity → love and care for nature → attitudes → intention → energy-saving behaviors	0.0014*	0.0008	[0.0003, 0.0037]	0.0014*	0.0008	[0.0003, 0.0037]
Physical activity → love and care for nature → subjective norms → intention → energy-saving behaviors	0.0013*	0.0008	[0.0003, 0.0035]	0.0013*	0.0008	[0.0003, 0.0035]
Physical activity → love and care for nature → perceived behavioral control → intention → energy-saving behaviors	0.0020*	0.0011	[0.0004, 0.0050]	0.0020*	0.0011	[0.0004, 0.0049]
Total indirect effect	0.0047*	0.0025	[0.0009, 0.0110]	0.0047*	0.0025	[0.0008, 0.0108]

*b* = Unstandardized indirect effect; *β* = Completely standardized indirect effect; *SE* = Standard error; BC = Bias corrected; CI = Confidence interval. Number of bootstrap samples for bias-corrected bootstrap confidence intervals: 10,000. *p < 0.05.

As Table 2 and Figures 2A–C illustrate, in all of the three models: The PA significantly and positively predicted students’ love and care for nature (M_1_), which in turn significantly and positively predicted students’ attitudes toward eco-friendly behavios (M_2_), subjective norms (M_3_), and PBC (M_4_). Students’ attitudes toward eco-friendly behaviors (M_2_), subjective norms (M_3_), and PBC (M_4_), each significantly and positively predicted students’ intention to perform eco-friendly behaviors (M_5_). Students’ PBC to perform eco-friendly behaviors was the strongest predictor of students’ intention to perform eco-friendly behaviors (M_5_), followed by students’ attitudes (M_2_). Lastly, students’ intention to perform eco-friendly behaviors (M_5_) significantly and positively predicted each of the three eco-friendly behaviors subscales (Y).

Table 3 depicts the indirect effects (specific and total) of the students’ PA to eco-friendly behaviors subscales via the serial-parallel mediators: love and care for nature (M_1_), attitudes (M_2_), subjective norms (M_3_), PBC (M_4_), and intention to perform eco-friendly behaviors (M_5_). As can be seen in Table 3 and Figures 2A–C, in all of the three models: The specific indirect effect from the students’ PA to the respective eco-friendly behaviors subscale through love and care for nature (M_1_), attitudes toward eco-friendly behavior (M_2_), and intention to perform eco-friendly behavior (M_5_) was statistical significant, as were also the specific indirect effect from the students’ PA to the respective eco-friendly behaviors subscale through love and care for nature (M_1_), subjective norms (M_3_), and intention to perform eco-friendly behavior (M_5_), and the specific indirect effect of the students’ PA to the respective eco-friendly behaviors subscale via love and care for nature (M_1_), PBC (M_4_), and intention to perform eco-friendly behavior (M_5_). Lastly, the total indirect effect from the students’ PA to the respective eco-friendly behavior’s subscale was also statistically significant.

### Covariate

In all of the three models, gender significantly predicted (positively) students’ love and care for nature and subjective norms to save energy. Particularly, girls reported higher scores in love and care for nature and in subjective norms to perform eco-friendly behaviors compared to boys. Also, in the serial-parallel mediation models linking the students’ PA to PA related energy-saving behaviors and energy-saving behaviors, gender significantly predicted (positively) students’ PBC to perform eco-friendly behaviors. More specifically, girls reported higher scores in PBC to perform eco-friendly behaviors compared to boys.

## Discussion

This study provides an interesting perspective on the exploration of the pathways linking PA and love for nature with eco-friendly behaviors in children, emphasizing the critical role of attitudes, subjective norms, PBC, and intentions as mediators in shaping this relationship. By examining these mediating variables, the study reveals how attitudinal factors influence children’s environmental actions, while also offering new insights for promoting environmental protection. The results of the present study highlighted the connection between students’ involvement in physical activities and their love and care for nature, as well as the utility of the TPB as a framework for understanding students’ eco-friendly behaviors. Attitudes, subjective norms, and PBC explained a significant proportion of the variance in students’ intentions to engage in eco-friendly behaviors. In turn, these intentions, effectively predicted environmentally conscious behaviors and energy-saving actions related to physical activities.

The ongoing discussion underscores the importance of fostering a positive and connected relationship with nature, which has been shown to support environmental attitudes. In fact, developing a meaningful bond with the natural world is increasingly recognized as a fundamental component of personal wellbeing. While research exploring the link between PA and emotional connections to nature remains limited, existing evidence suggests that walking interventions particularly those involving intentional, nature-focused activities can significantly deepen individuals’ sense of connection to nature, even within built environments (Pasca et al., 2020; Lumber et al., 2017). Furthermore, a growing body of research highlights the substantial emotional and psychological benefits that natural environments provide. Exercising in natural setting and walking in nature, in particular, stands out as a simple yet powerful form of PA that promotes emotional wellbeing and improves mood (Olafsdottir et al., 2020; Yao et al., 2021). Moreover, a relevant study indicated that attitudes played an especially important role with respect to the two forms of active mobility, walking and cycling (Stark et al., 2025).

The current study additionally revealed an interesting sequence of relationships: higher levels of PA were associated with greater love for nature, which in turn was linked to the development of positive attitudes toward environmental protection. Particularly, love and care for nature significantly influenced their general attitudes toward energy conservation, as well as the social pressure they perceived to engage in such behaviors. Additionally, it contributed to students’ sense of control over their ability to save energy. The strong impact in this study of love and care for nature on attitudes, subjective norms, and PBC is noteworthy. These findings provide useful information for interventions designed to encourage eco-friendly behaviors by means of increasing people’s love and care to nature.

Despite the above sequence of relationships, it is important to note, that it is plausible that the examined links reflect bidirectional-circular relationships (Bernard et al., 2021). Students’ engagement in current eco-friendly behaviors could enhance their intention to perform eco-friendly behaviors in the future, which in turn may also strengthen their attitudes, subjective norms, and PBC toward eco-friendly behaviors. Similarly, enhancing students’ attitudes, subjective norms, and PBC toward eco-friendly behaviors may also lead to a stronger love for nature, which in turn may lead students to participate in more PA.

Attitudes toward eco-friendly behaviors, subjective norms, and PBC, emerged as significant predictors of individuals’ intentions to engage in environmental actions across all three models developed in the present study. Consistent with principles of the TPB, these findings underscore the importance of both individual cognitive evaluations and perceived social influences in shaping behavioral intentions. Moreover, behavioral intention was found to contribute meaningfully to the prediction of actual eco-friendly behaviors, encompassing general energy-saving practices as well as those specifically related to PA.

Overall, these results highlight the role of intention as a proximal determinant of behavior. The three factors, attitudes, subjective norms, and PBC each had a significant and positive influence on students’ intentions to engage in eco-friendly behaviors. Among them, PBC was the strongest predictor of intention, followed by attitudes. Moreover, students’ intention to engage in eco-friendly behaviors significantly predicted all three categories of eco-friendly actions examined in the study. These findings are consistent with those of other related studies that, using the ΤPB, have explored the relationships between attitudes and energy-saving behaviors (de Leeuw et al., 2015; Ojala, 2012). Studies have shown that individuals living closer to inland blue spaces tend to be more physically active, experience higher levels of wellbeing, and report lower symptoms of depression and anxiety (Roba et al., 2025; Murrin et al., 2023). Additionally, a significant correlation has been identified between PA and pro-environmental behavior (Zhou et al., 2025). Building on these findings, the present study revealed an important indirect relationship between students’ PA and their eco-friendly behaviors. This relationship is fully mediated by students’ love and care for nature, attitudes toward sustainable practices, subjective norms, PBC, and their intentions to engage in environmentally responsible actions. The identified direct and indirect effects linking PA to eco-friendly behaviors, highlight the significance of incorporating climate change awareness and the adaptive benefits of PA into Physical Education curricula. Given the dual role of active transportation—as both a strategy for mitigating climate change and a means of enhancing PA it is imperative to develop and implement targeted interventions that promote its widespread adoption. In this context, the disciplines of exercise and sport psychology are uniquely positioned to contribute to addressing the urgent challenges posed by climate change. These fields can play a critical role in designing psychologically informed strategies that encourage sustainable behavior change and in transforming PA and sport practices into environmentally responsible and carbon-neutral endeavors. In doing so, they support broader efforts to mitigate the detrimental effects of climate change while promoting public health and wellbeing (Bernard et al., 2021; Bernard et al., 2024).

Gender differences also emerged. In all examined models, compared to boys, girls demonstrated stronger feelings of love and care for nature, as well as more favorable subjective norms regarding eco-friendly behavior. Also, in the serial-parallel mediation models linking the amount of students’ PA to PA related energy-saving behaviors and energy-saving behaviors, girls reported higher scores in PBC to perform eco-friendly behaviors compared to boys. It is widely recognized that younger generations exhibit a higher level of engagement with climate change issues compared to older cohorts (Poortinga et al., 2023). This raises an important question: at what stage of development are environmentally responsible attitudes most effectively formed? Environmental attitude and behavior begin to develop in early childhood, typically emerging around the age of 7. These traits tend to increase until around age 10, stabilize through age 14, and then gradually decline (Otto et al., 2019). Given that the present study assessed attitudes and environmental behaviors across these age groups, its findings are particularly valuable. They highlight the importance of targeting intervention programs at these critical developmental stages—through activities that foster physical engagement, nurture a connection with nature, and implement effective attitude-shaping strategies. Relevant school programs that are integrated into physical education modules offer an excellent opportunity to raise awareness about environmental issues.

### Practical implications

Public health researchers, policymakers, and practitioners have long recognized the influence of built environments on health outcomes and health-promoting behaviors such as walking (Brown et al., 2023). The present findings suggest that intervention strategies should extend beyond promoting PA alone to also encompass components that cultivate emotional connection to nature and outdoor environment and foster the development of pro-environmental attitudes. Integrating these elements into public health and educational initiatives may support individuals in adopting more sustainable, eco-friendly behaviors. For example, it was suggested school-based programs that promote active transportation, like walk-or-bike-to-school days, and everyday choices, such as taking the stairs instead of the elevator, both at school and at home, can help save energy and reduce carbon emissions while empowering students through PA (Zhou et al., 2025). Integrating short, planned “green exercise” breaks (e.g., energizing activities combined with environmental messages about nature) can strengthen the connection between personal wellbeing and environmental responsibility. Activities involving parents or peers can further boost motivation and social support, with parental involvement playing a key role. Community-based events, such as those focused on nature, and exercising in green and blue environments can extend these positive behaviors beyond the school environment.

Furthermore, of particular importance is the need to engage younger populations, encouraging them to internalize these values, and providing opportunities for them to experience positive emotions through behaviorally activating activities. Through movement-based activities, students can develop a deeper emotional connection to nature while cultivating core environmental values. Integrating PA with environmental education and the promotion of pro-environmental attitudes within schools and public health initiatives offers a promising, multidimensional strategy for fostering lasting environmental sensitivity and sustainable behaviors. Moreover, increasing educational knowledge and exposure to information about climate change has been consistently linked to higher levels of pro-environmental behavior, underscoring the importance of incorporating these themes into broader educational programming (Vrselja et al., 2024).

Self-control enables individuals to align their behaviors with their personal attitudes, indicating that it is a pivotal psychological trait for safeguarding long-term pro-environmental goals (Wyss et al., 2022). Accordingly, this factor should be carefully considered in the design and implementation of environmental awareness education programs (Otto et al., 2019). A related study underscores the essential role of physical education in advancing environmental sustainability, recommending that policymakers prioritize the development of educational initiatives integrating sustainability principles and technological innovations. These programs should highlight the intricate relationship between human wellbeing and the natural environment, thereby acting as a catalyst for cultivating environmentally responsible attitudes and behaviors among participants (Liu et al., 2024).

The results indicate that girls exhibit higher levels of sustainability awareness than boys. These findings are in line with the results of a relative review by Gifford and Nilsson (2014), who found that women tend to express higher environmental concern and stronger intentions for pro-environmental actions than men, although they often score lower on factual environmental knowledge tests. These disparities may be linked to gender socialization, including women’s tendency towards other-orientation, altruistic concerns, and potential historical educational disparities in science and environment fields (Gifford and Nilsson, 2014). Overall, these findings underscore the importance of designing educational interventions that are inclusive and responsive to gender-based differences, in order to effectively promote pro-environmental behaviors among all students. Such targeted approaches are essential not only for strengthening students’ environmentally responsible practices but also for advancing broader goals of social equity and sustainable development. Consequently, addressing gender disparities in environmental awareness and engagement constitutes a critical step toward the construction of fairer, healthier, and more resilient societies (Boned-Gómez et al., 2025).

### Limitations

One potential limitation of this study lies in its reliance on self-reported measures of eco-friendly behaviors. While obtaining objective data for the wide array of behaviors assessed would have been impractical, the use of self-report is consistent with standard practice in most research on pro-environmental behavior. To mitigate potential biases, participants were assured of the anonymity of their responses, the sample size was substantial, and a diverse range of eco-friendly behaviors was examined, all factors that contribute to the robustness of the findings. Moreover, many of the results align with those of previous studies investigating similar variables (de Leeuw et al., 2015), lending additional support to their validity. Beyond the limitations of self-report, it should also be noted that the cross-sectional design of this study precludes causal inferences. Additionally, as the research was conducted within a Greek cultural context, the generalizability of the findings to other cultural settings may be limited, underlining the importance of replication in diverse populations.

### Conclusion

In conclusion, this study offers several important contributions to the growing body of research on eco-friendly and energy-saving behaviors. The proposed conceptual framework integrates a diverse range of meaningful variables, including PA, emotional connection to nature, and both attitudes and intentions regarding pro-environmental and energy-conserving actions. Notably, the mediation model identified a potential role for love of nature and positive attitudes, subjective norms, PBC, and intention toward environmental practices in mediating the relationship between physical activity and eco-friendly behaviors. Furthermore, the age group represented in this sample is at a particularly formative developmental stage, making it especially important to foster positive attitudes toward exercise, nature, outdoor activities, and environmental responsibility. Future longitudinal and experimental studies examining the causal relationships among these relevant factors would greatly enrich our understanding of the mechanisms underlying pro-environmental behaviors. Additionally, cross-cultural replications are essential to assess the generalizability of these findings across different sociocultural contexts. Building on these insights, future research can also draw upon the key beliefs and variables identified in this study to inform the development of targeted educational interventions. Such programs could play a vital role in cultivating environmental awareness and responsible behaviors among children and adolescents, thereby contributing to broader efforts toward sustainable development.

## Data Availability

The raw data supporting the conclusions of this article will be made available by the authors without undue reservation.
