# Supplementary material for: Exploring the pathways between physical activity, love for nature and eco-friendly behavior in children
Source: Front Psychol. 2026 Jan 12;16:1710555. doi: 10.3389/fpsyg.2025.1710555 (PMC12832462; doi:10.3389/fpsyg.2025.1710555)
Supplement: Supplementary file 1 [file Supplementary_file_1.pdf]

Supplementary file 1. Measures.

Concept	Items
Physical Activity.	On a typical week how many hours per week (from 0 to 7 h) in your free time engaged in physical activity so much that you get out of breath or sweat. 8-point Likert scale from 0 (0 hours) to 7 (7 hours per week).
Love and Care for Nature.	I feel joy just being in nature; I feel that closeness to nature is important for my wellbeing; When I am close to nature, I feel a real sense of oneness with nature; I feel content and somehow at home when I am in unspoilt nature; I feel a deep love for nature; I often feel emotionally close to nature; When I spend time in unspoilt nature I feel that my day-to-day worries seem to dwindle away in the face of the wonder of nature; Protecting the wellbeing of nature for its own sake is important to me; I feel spiritually bound to the rest of nature; I feel a personal sense of interconnectedness with the rest of nature; I often feel a sense of awe and wonder when I am in unspoilt nature; I often feel a strong sense of care towards the natural environment; I need to have as much of the natural environment around me as possible; When in natural settings I feel emotionally close to nature; I enjoy learning about nature: 7-point Likert-type scale: 1 *(strongly disagree*) to 7 *(strongly agree*).
Attitudes	For me, performing eco-friendly behaviors on a regular basis during the next 6 months would be: Five 7-point bipolar adjective scales “useless-useful,” “unpleasant-pleasant,” “good-bad,” “happy-sad,” and “pretty-ugly”.
Perceived Behavioral Control.	For me, performing eco-friendly behaviors on a regular basis in the next 6 months would be : 7-point scale: “very difficult” to “very easy”. “I feel that I'm able to perform pro-environmental behaviors on a regular basis in the next 6 months”: 7-point scale: “definitely not” to “yes, definitely”.
Subjective Norms.	In general, people who are close to me expect me to adopt eco-friendly behaviors on a regular basis during the next 6 months; People who are important to me will perform eco-friendly behaviors on a regular basis during the next 6 months: 7-point scale “definitely not” to “yes, definitely”.
Intentions.	I intend/I will try/ I am determined to perform eco-friendly behaviors on a regular basis during the next 6 months: 7-point scale: “definitely not” to “yes, definitely”.
Eco-Friendly Behaviors.	Pro-environmental behaviors: I leave the water running while I brush my teeth; I forget to turn off the light when I leave my room to go eat; I leave the fridge door open while I think about what I go eat; At home, I put my trash in the proper recycling bin; At school, I put my trash in the proper recycling bin; I leave the TV on while I'm doing other things in the house; I turn off the TV or the video game when I go eat; I shower for more than 20 min; When I'm cold, I put on a sweater instead of turning up the heat; I consume biological products: 7-point scale: never-always.
Physical Activity-Related Energy-Saving Behaviors:	I go to school by bike or on foot; In general, for short distances, I travel by car or by bus; At home, I prefer to use the stairs instead of the elevator; In general, for short distances, I prefer to walk, both in winter and summer; In general, for short distances, I avoid using a car or a bus: 7-point: Likert-type scale: never to very often.
Energy-Saving Behaviors:	I switch off the lights even if I’m not using them for a short time; Even if I didn’t turn on the lights or electrical appliances, I switch them off when they are not in use; I do not switch on the lights if natural light is sufficient; If the weather is good, I open the windows instead of turning on an air conditioner or a fan: 7-point Likert-type scale: never to very often.
